# Infected Leg Hematoma: A Rare Cause of Recurrent Leg Cellulitis

**DOI:** 10.7759/cureus.18459

**Published:** 2021-10-03

**Authors:** Emmanuel J Nebuwa, Sadaf Rahman, Uchenna Okoye, Ugochi Ojinnaka

**Affiliations:** 1 Internal Medicine, Rockville Ambulatory Surgery Center, Silver Spring, USA; 2 Internal Medicine, Anne Arundel Medical Center, Annapolis, USA; 3 Intensive Care Unit, Adventist Health System, White Oak, USA; 4 Family Medicine, Lankenau Medical Center, Wynnewood, USA

**Keywords:** recurrent bacteremia, sepsis, anticoagulant therapy, cellulitis, hematoma

## Abstract

Intramuscular hematoma most commonly occurs following trauma; however, spontaneous hematomas may be seen in elderly patients due to anticoagulation. Intramuscular hematomas may not be chronically expanding, and their signs and symptoms vary, ranging from asymptomatic to swelling that may be expanding in size. In some cases, chronic hematoma may become infected as we witnessed in the case we present in this report. Our patient reported recurrent cellulitis and occasional fever with no other history of probable cause of the recurrent cellulitis. Such an atypical presentation poses a risk of delayed or missed diagnosis of a condition that can threaten the limb of a patient, as seen in our case.

## Introduction

While traumatic muscular hematomas can occur in patients of all demographics, spontaneous muscle hematoma (SMH) is more commonly associated with the elderly population receiving treatment with anticoagulants; it is reported to occur in approximately 5% of such patients with an annual mortality rate of 0.65% [1]. SMH is an uncommon condition that is often overlooked or misdiagnosed [2], and it is potentially life-threatening, particularly in elderly and frail patients [3]. Several predisposing or contributing factors have been described, and the most frequent ones include minor trauma, increased abdominal pressure, anticoagulation medications, hypertension, and iatrogenic causes [3]. SMH has a significantly increased incidence in the elderly and frail patients receiving treatment with anticoagulants [4]. In the majority of cases, muscle hematomas affect the abdominal rectus sheath or the gluteal muscles. Lower extremities like the calf are an uncommon site of hematoma [3]. Therefore, a high degree of suspicion is always needed when patients present with pain, edema, and ecchymosis in a lower extremity muscle region [5]. Infected spontaneous hematoma of the lower extremities is very rare and currently, there is no data in the literature on this mysterious entity. In this report, we present the case of a chronically ill elderly patient who developed a non-traumatic intramuscular hematoma of the leg muscle associated with apixaban therapy.

## Case presentation

A 64-year-old male with a past medical history of atrial fibrillation on apixaban presented to the ER in a febrile condition complaining of right leg pain with swelling and serosanguineous drainage. He had initially visited the urgent care facility a few weeks ago for the management of leg pain with erythema and had been treated with antibiotics. His symptoms had reoccurred after two weeks, which had led him to present back to the ED due to the recurrent cellulitis associated with fever, chills, and wound abscess.

**Figure 1:**
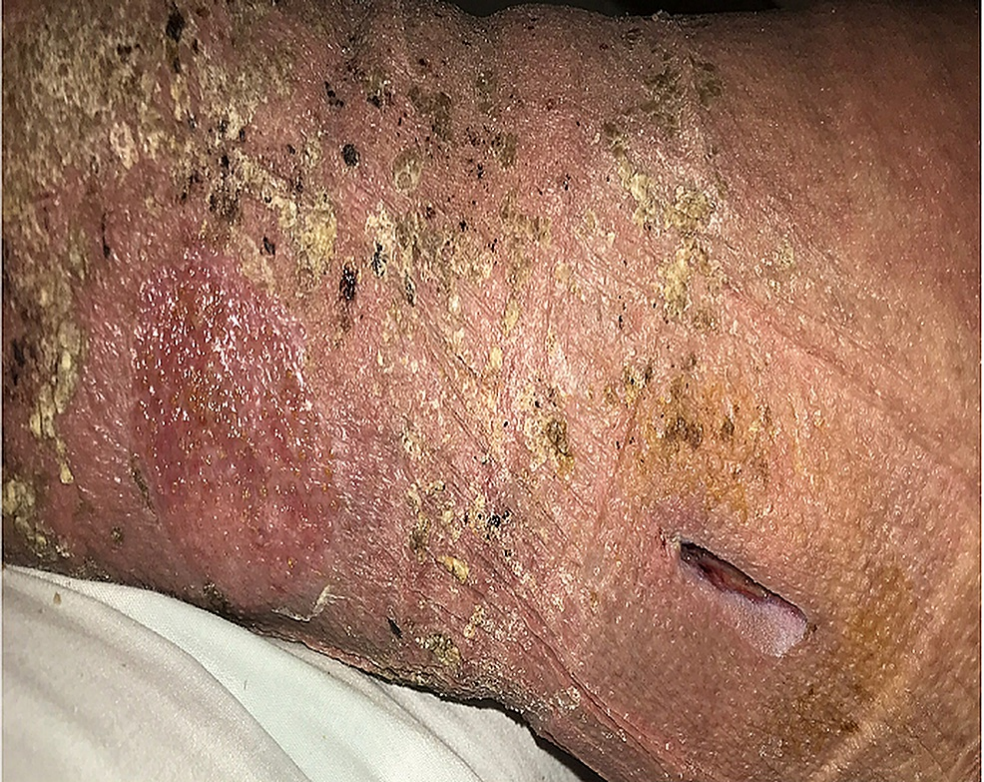
A picture of the cellulitis and associated open wound

A review of systems showed fever, drowsiness, genialized weakness, mild confusion, nausea, vomiting, and bilateral lower extremity skin rash. There was no associated joint pain or swelling, dysuria, chest pain, or abdominal pain. Examination showed dry skin with hyperkeratotic plaques with peeling and bilateral lower extremity non-pitting edema. Right medial calf open wound was noted and was draining minimal serous fluid with some erythematous streaking, and granulation tissue (Figure 1).

**Figure 2:**
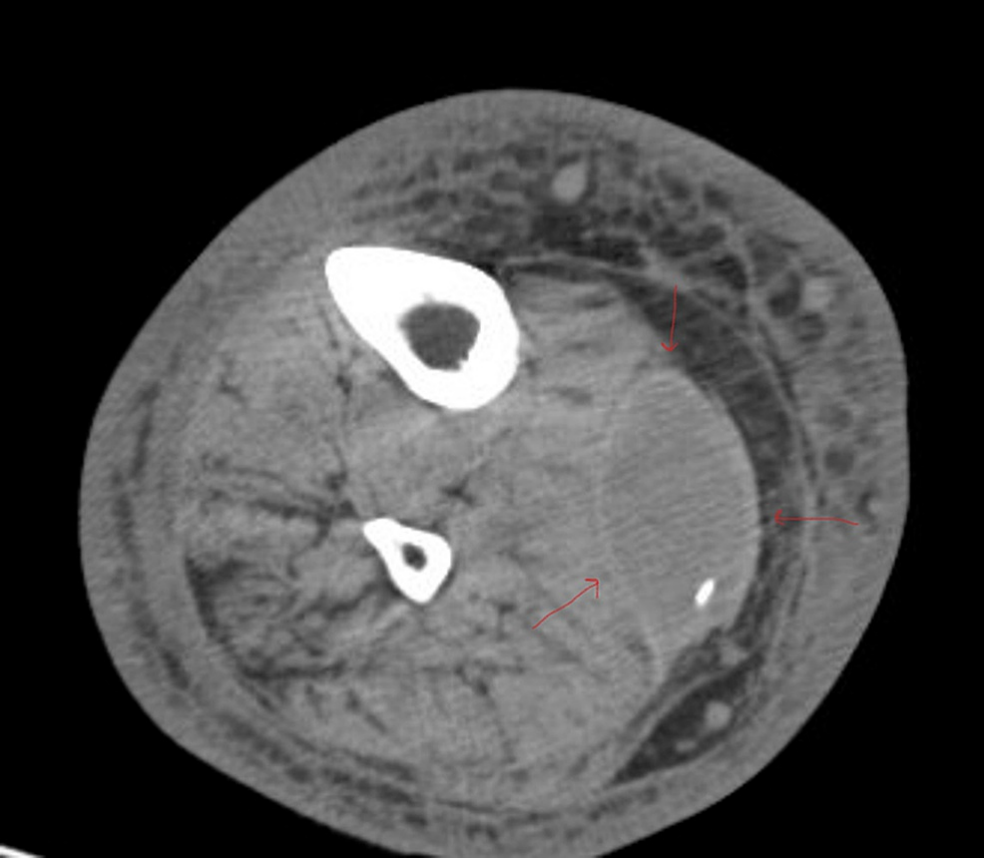
CT scan showing the intramuscular hematoma CT: computed tomography

The patient was admitted on account of recurrent cellulitis for further evaluation. On admission, he had a temperature of 98.1 °F, a heart rate of 102 beats per minute, a blood pressure of 140/61 mmHg, and an elevated respiratory rate (18). Laboratory workup was done, which showed a white blood cell count of 3.26 x 10^9^/L, hemoglobin (Hb) of 10.8 g/dL, c-reactive protein (CRP) of 10.2 mg/L, and procalcitonin of 0.13 ng/ml. The culture of the wound swab showed no growth, and the same was the case with blood culture, acid-fast bacilli (AFB) wound culture, as well as urine culture and reflex culture. Methicillin-resistant *Staphylococcus aureus* (MRSA) nasal was negative, and so were respiratory panel and C. diff toxin. The liver function test (LFT) was within the normal range. Chest X-ray showed no acute infectious process. Transthoracic echo was negative for obvious vegetations. The patient was empirically started on IV vancomycin and Zosyn. A series of imaging was done to find the source of the infection. CT angiography (CTA) of the chest was ordered and came back negative for pulmonary embolism (PE) or consolidation. CT of the abdomen and pelvis revealed ileus enteritis but no abscess. CT tibia and fibula right with IV contrast (Figure 2) showed findings consistent with right lower extremity cellulitis, with a lentiform-shaped collection situated between the soleus and gastrocnemius muscles, which are most suggestive of a hematoma. An incision and drainage were done to drain the abscess and the hematoma. Repeat culture of blood came out positive for *Serratia marcescens* resistant to amoxicillin/clavulanate and sensitive to piperacillin/tazobactam. He was started on a four-week course of IV ertapenem and was discharged home with a peripherally inserted central catheter (PICC) line to continue the antibiotics and advised to follow up with outpatient wound care.

The patient reported that he was doing well until the discontinuation of ertapenem, following which he had recurrent pain and swelling in the right lower leg accompanied by fever and chills. His condition significantly worsened within a few days. He returned to the ED and was admitted on account of recurrent cellulitis. Laboratory workup showed leukocytosis and blood culture grew *Serratia marcescens*. A repeat CT of the leg with IV contrast showed a persistent hematoma, similar to the previous CT. The hematoma was suspected as the nidus for bacteremia and ongoing infection. Transesophageal echo showed no vegetation. He was referred to another facility where he had previously received care for incision and drainage of the abscess with some of the hematoma evacuated. During the incision and drainage, a 15-cc sanguinopurulent drainage was drained and culture showed growth for *Serratia marcescens*. He was continued on IV ertapenem during the hospitalization, with a plan to continue wound care and repeat blood culture to ensure the eradication of bacteremia before discharge. The blood culture came back with no growth and he was discharged home on Bactrim and advised to follow up with cardiology for his anticoagulation.

However, after one month, the patient presented back to the ED with sudden onset of fever and altered mental status with the right lower extremity cellulitis. He was admitted and an extensive workup for the source of bacteremia was done. No evidence of infection was noted. The likely source was assumed to be the intramuscular hematoma in the right lower extremity that measured 5.6 x 3.5 cm on a repeat CT scan. He underwent an incision and drainage of the right leg abscess and subsequent evacuation of the hematoma. The postoperative report showed intramuscular chronic abscess with thick pseudo-capsule and cheese-curd-like material in the location of the hematoma. The culture of the hematoma yielded growth for *Serratia marcescens*. Postoperatively, he remained hemodynamically stable. He was monitored overnight and subsequently discharged home on IV ertapenem via PICC line for four weeks. The patient was advised to follow up with weekly labs, and a follow-up visit with infectious disease upon completion of antibiotics was also recommended.

## Discussion

The present case shows that recurrent lower extremity cellulitis secondary to infected intramuscular hematoma in elderly patients could be easily missed under a low level of suspicion.

We discussed the case of an elderly patient with lower extremity cellulitis who presented to the ED and was discharged home on antibiotics with a recommendation to revisit the ED upon the onset of any symptoms. The patient developed a non-healing wound within two weeks after hospital discharge, necessitating a re-admission for further evaluation.

This case is the first to report an infected non-expanding intramuscular hematoma with resultant recurrent leg cellulitis following atraumatic leg pain and swelling. In the literature, only a few cases of lower extremity hematoma have been associated with localized infection [6]. However, most cases reported are of chronically expanding hematoma secondary to trauma or anticoagulant therapy [7].

In addition, we reported the unique finding of intramuscular hematomas, leading to multiple incisions and drainage and recurrent sepsis. Isolated non-expanding hematoma between muscle groups in the leg is a rare occurrence and poses a serious threat to the limb when infected. For a quick diagnosis and to choose the best treatment, a high degree of suspicion is needed especially in patients with acquired coagulopathy [3]. As also reported by Baek and Kim [8], morbidity due to infected hematoma in the lower extremity can also be avoided if a high level of suspicion is maintained [3].

Our patient took apixaban for atrial fibrillation prophylaxis, which may have contributed to his hematoma formation in the lower extremity [3]. On the culture of the hematoma, after incision and drainage in the OR with subsequent hematoma evacuation, a growth of *Serratia marcescens* was noticed. This organism is a gram-negative bacillus that occurs naturally in soil and water and produces a red pigment at room temperature. It is associated with urinary and respiratory infections, endocarditis, osteomyelitis, septicemia, wound infections, eye infections, and meningitis. However, it is not one of the most common causes of hematoma infections [9].

Our case illustrated the difficulties in diagnosing and treating recurrent cellulitis in a geriatric patient. Although our patient eventually started recuperating well, it is important to realize that an infected intramuscular hematoma is a potential cause of recurrent sepsis [10]. Therefore, we recommend having a high level of suspicion when an older patient on anticoagulant presents to the ED with non-healing cellulitis, followed by recurrent sepsis. It is pertinent to always consider an infected intramuscular hematoma especially in a patient on anticoagulation therapy.

## Conclusions

We discussed a rare case of recurrent cellulitis due to an infected intramuscular hematoma. This report highlights the importance of including suspected hematoma on the differential list, especially in elderly patients on anticoagulation. Physicians need to be vigilant of atypical presentation of infected hematoma and consider a multidisciplinary approach in any patient having recurrent cellulitis and bacteremia of an unknown source.
